# Microbiopsy engineered for minimally invasive and suture-free sub-millimetre skin sampling

**DOI:** 10.12688/f1000research.2-120.v2

**Published:** 2013-07-31

**Authors:** Lynlee L Lin, Tarl W Prow, Anthony P Raphael, Robert L Harrold III, Clare A Primiero, Alexander B Ansaldo, H Peter Soyer

**Affiliations:** 1 Dermatology Research Centre, The University of Queensland, School of Medicine, Translational Research Institute, Brisbane, QLD 4012, Australia

## Abstract

We describe the development of a sub-millimetre skin punch biopsy device for minimally invasive and suture-free skin sampling for molecular diagnosis and research. Conventional skin punch biopsies range from 2-4 mm in diameter. Local anaesthesia is required and sutures are usually used to close the wound. Our microbiopsy is 0.50 mm wide and 0.20 mm thick. The microbiopsy device is fabricated from three stacked medical grade stainless steel plates tapered to a point and contains a chamber within the centre plate to collect the skin sample. We observed that the application of this device resulted in a 0.21 ± 0.04 mm wide puncture site in volunteer skin using reflectance confocal microscopy. Histological sections from microbiopsied skin revealed 0.22 ± 0.12 mm wide and 0.26 ± 0.09 mm deep puncture sites. Longitudinal observation in microbiopsied volunteers showed that the wound closed within 1 day and was not visible after 7 days. Reflectance confocal microscope images from these same sites showed the formation of a tiny crust that resolved by 3 weeks and was completely undetectable by the naked eye. The design parameters of the device were optimised for molecular analysis using sampled DNA mass as the primary end point in volunteer studies. Finally, total RNA was characterized. The optimised device extracted 5.9 ± 3.4 ng DNA and 9.0 ± 10.1 ng RNA. We foresee that minimally invasive molecular sampling will play an increasingly significant role in diagnostic dermatology and skin research.

## Introduction

**Figure 1.**
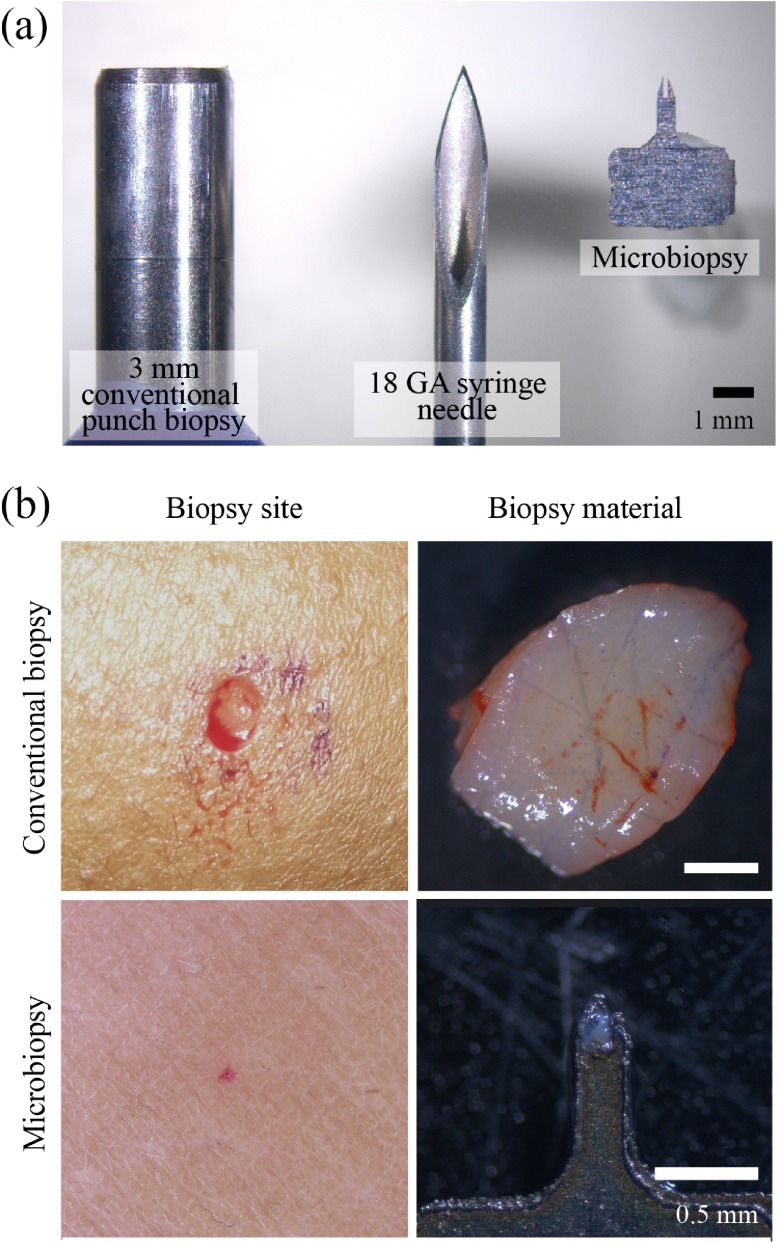
Size comparison of needle, biopsy devices and biopsy comparisons. A conventional biopsy punch is shown on the left, an 18 gauge syringe needle in the centre and the inner chamber of our microbiopsy device on the right Panel ( **a**) our microbiopsy device chamber is 0.15 mm in width with an outer width of 0.25 mm. The top row of Panel ( **b**) contains a conventional 3 mm biopsy site and tissue, whereas the bottom panels show microbiopsied skin and tissue.

Skin biopsy is one of the most essential techniques in dermatology for accurate diagnosis of neoplastic or inflammatory skin diseases through histopathological assessment. This technique is performed under local anaesthetic by trained medical personnel, normally a dermatologist, to remove a skin sample 2–4 mm in diameter ( Figure 1) that is then preferably sent to a dermatopathologist for histopathological diagnosis. Pathological examination using skin biopsies often complements and/or confirms diagnosis of common neoplastic or inflammatory skin diseases ^1^. This technique is usually performed using a punch biopsy tool or a scalpel. The wound is then either sutured or left to heal.

An excisional biopsy, a common alternative in neoplastic skin conditions, is performed when the entire tumour is needed for histopathological examination, or when melanoma is suspected. This depends on the size and location of the lesion and can be performed using a larger 4–6 mm diameter punch biopsy, a deep shave biopsy, or with a fusiform excision ^2^. These biopsy techniques coupled with histopathology are able to provide accurate diagnosis of the disease occurrence and progression, however the downsides of these techniques are that they require local anaesthesia and sutures in addition to the time required to carry out the procedure. Furthermore, samples are generally fixed with formalin that hinders molecular analysis. Molecular fingerprinting of skin disease has the potential to dramatically improve diagnostic sensitivity and open the door for personalised medicine ^3– 5^. To date, there have been no reports on biomarker profiling of lesional samples in a prospective study due to the lack of suitable technologies to perform multiple sampling over time. This is due to the iatrogenic issue of cutting out the lesion through biopsy precluding further study of that lesion.

This problem exists in many medical disciplines and has led to the evolution of experimental diagnostic devices towards miniaturised versions of their predecessors. This miniaturisation trend is enabled by several factors including improved micro-manufacturing tolerances, decreasing costs and increasing availability. One of the earliest micro-devices developed to obtain biopsy samples was patented by Krulevitch *et al.* ^6^. They patented microbiopsy/precision cutting devices fabricated by conventional machining, silicon micromachining, precision machining and injection moulding. Over the years, there have been many similar patents that report a variety of such microbiopsy devices. The unifying theme is that these patents all describe micro-biopsy devices for breast or intestinal tissue sampling ^7– 10^ and importantly none were engineered for skin or skin lesions.

Our group has developed a new microbiopsy platform technology that enables the collection of tiny pieces of skin using a micro-medical device that is minimally invasive and does not require local anaesthesia (IP Australia Appl. Num. 2012901490, filed on 16/04/2012). There are situations when conventional biopsies are not appropriate. Skin disease may present with multiple lesions and/or in a cosmetically sensitive area. The microbiopsy device has the potential to fill a void in dermatology where conventional biopsies are not feasible or could do more harm than good. We have observed that microbiopsy sampling does not interfere with downstream histopathological diagnosis ^11^. The data presented herein describe the fabrication, optimisation and early steps toward the validation of a novel microbiopsy device for use *in vivo*.

## Materials and methods

### Microbiopsy device fabrication

The microbiopsy devices used in our studies were fabricated by laser cutting plates of stainless steel. The in-house fabrication of microbiopsy devices involves laser cutting of two-dimensional designs on 0.05 mm thick medical grade stainless steel (304, Mastercut Technologies, Australia) at 95% power, 30% frequency and speed of 0.9 using a 20 W laser marking system (LaserPro S290, GCC, Taiwan). All of the 2D microbiopsy components were assembled into 3D devices, and sterilized using a glass bead sterilizer (Steri Inotech 350, Sigma-Aldrich, USA) at 250°C for 30 seconds or submerged in 70% ethanol for 1 min and air-dried prior to use. The microbiopsy device was then fitted into a spring-loaded applicator.

### Microbiopsy device imaging

A bench top scanning electron microscope (SEM) (JCM-6000 Neoscope, JEOL, USA) was used to acquire high-resolution images of the microbiopsy device. The roughness amplitude of the microbiopsy chamber was obtained by measuring the average distances of the edge to a regression-fitted straight line using MatLab (Mathworks, Australia).

### Microbiopsy and conventional biopsy sample collection

Microbiopsy samples were either obtained *in vivo* from healthy volunteers (HREC/12/QPAH/082, Metro South Human Research Ethics Committee, Centres for Health Research, Princess Alexandra Hospital) or *ex vivo* from excised actinic keratosis (AK) lesions. The skin of a healthy volunteer was swabbed with alcohol and the microbiopsy applied. Conventional biopsies e.g. punch ( Figure 1b) or shave biopsies, were performed with informed consent from patients (HREC/08/QPAH/207 for healthy skin or HREC/11/QPAH/477 for AK lesions) prior to being microbiopsied. Samples were collected in sterile RNase and DNase free 1.5 ml microcentrifuge tubes containing either RNALater ^®^ (Life Technologies, USA) or pH 7.0 phosphate buffered saline (PBS) on ice within 20 minutes after tissue removal from patients. All samples placed in RNALater ^®^ were kept overnight at 4°C and then stored at -80°C.

### Measurement of pain score

Volunteer pain scores were evaluated with Metro South Human Research Ethics Committee, Princess Alexandra Hospital approval (HREC/12/QPAH/082). Each of the 20 volunteers ( Table 1) was presented with an assessment form to grade his/her expected pain score based on a numerical rating 10-point Likert scale, 0 as having no pain and 10 as pain as bad as they can imagine before the start of the experiment. Each microbiopsy application was performed one minute apart and the pain score recorded immediately after application. Five minutes after the final microbiopsy application, the volunteers were asked to rate the level of pain for each microbiopsy site.

**Table 1.** Volunteer summary. Volunteers for microbiopsy classified by age, ethnic group, gender and Fitzpatrick skin type.

Ethnic group	Fitzpatrick skin type ^22^	Gender	Age (as of study year)	Number of participants
Caucasian	II–III	M	23–44	8
Caucasian	I–II	F	22–39	6
Asian	IV	M	27–33	4
Asian	III	F	27	2

### Reflectance confocal microscopy

Microbiopsy sites were visualized with a reflectance confocal microscope (RCM, Vivascope 1500 ^®^ Multilaser, Lucid, Inc., USA) in volunteers or in excised skin (HREC/12/QPAH/217). RCM was also used to perform *in vivo* examination of the skin after microbiopsy. RCM images were collected through the microscope head, integrated with a water immersion objective, at a near-infrared wavelength of 785 nm. Blocks consisting of 7 × 7 mm mosaics of stitched RCM images and 2 µm to 200 µm vertical z-stacks were acquired at the microbiopsy sampling site. Dermatoscopic images were collected with a Canon Power Shot G10 digital camera (Canon, Japan) and a dermatoscope attachment (Dermlite©, 3Gen, USA).

### Microbiopsy site visualisation histology

Microbiopsied tissue was embedded (Optimal cutting temperature compound, Sakura Finetek, USA) and cryosectioned (CM1850, Leica Microsystems Pty Ltd, Australia). The 10 µm thick sections were fixed with 100% cold methanol for 10 minutes, air dried and stained with haematoxylin and eosin (Varistain Gemini ES, Thermo Fisher Scientific Inc., USA) to visualize the microbiopsy sites.

### Nuclear labelling and confocal microscopy

The microbiopsy extracted tissue was incubated with DRAQ5 (BioStatus Limited, UK) at 10 µM in PBS for 30 minutes at room temperature. Confocal microscopy images were taken with a Zeiss 510 META confocal microscope (Carl Zeiss Microscopy GmbH, Germany). A 3D projection was then created from the z-stacks (2 μm steps) of confocal images using Imaris (Bitplane Scientific Software, Switzerland). ImageJ (NIH, USA) was used to estimate the nuclei in microbiopsy samples.

### DNA isolation

DNA was extracted from the microbiopsy samples using QIAamp DNA Micro purification kits (QIAGEN GmbH, Germany) using a protocol modified to accommodate the unique microbiopsy sample. The microbiopsy was removed from the applicator housing after application. The presence of a tissue sample was quickly confirmed by opening the microbiopsy device and visualizing with a stereo microscope (Carl Zeiss Microscopy GmbH, Germany). The opened device was then immersed in 180 μl lysis buffer (Buffer ATL, QIAGEN GmbH, Germany) in the presence of proteinase K (20 μl) in a 1.5 ml tube. Fifteen seconds of pulse-vortexing was applied to ensure that the sample was well-mixed. The sample was then placed in a thermomixer (Eppendorf, Australia) at 56°C with shaking at 800 rpm overnight. The tube containing the device was briefly centrifuged for 10 seconds to remove the solution adhering to the cap of the tube before transferring the lysate into a new tube. The tube containing the device was centrifuged again at 6000 g for 30 seconds to remove any lysate adhering to the device. The lysates were combined and the remaining procedure followed the manufacturer supplied instructions.

### RNA isolation

The modified sample lysing processing described in DNA isolation above was also used with the Arcturus ^®^ PicoPure ^®^ RNA Isolation Kit (Life Technologies, USA) to obtain total RNA from microbiopsy samples. RNA isolation of lesional samples was performed using RNeasy Mini Kit (QIAGEN GmbH, Germany) according to the RNeasy Mini Handbook (version 2010).

### DNA and RNA quantification

A quantitative fluorometer-based assay (Qubit ^®^ 2.0, Life Technologies, USA) was used to determine the concentration of DNA and RNA with the protocol provided by the manufacturer.

### DNA and RNA quality control

Agilent DNA 12000 DNA kit (Agilent Technologies, USA) was used to determine the integrity and quality of DNA after whole genomic amplification of isolated DNA from microbiopsy and matched conventional shave biopsy samples. Agilent RNA 6000 Nano and Pico kits (Agilent Technologies, USA) were used to determine the RNA integrity number (RIN) of lesional and microbiopsy samples after RNA isolation. The supplied protocol was followed.

### Whole genomic amplification

Identical amounts of total DNA (1.85 ng) for both lesional and microbiopsy samples were subjected to whole genomic amplification. The amplification procedure was carried according to manufacturer's instructions (REPLI-g Single Cell Kit, QIAGEN, Australia).

### Whole transcriptome amplification

Identical amounts of total RNA (14 ng) for both lesional and microbiopsy samples were subjected to whole transcriptome amplification. The procedure was carried out using the supplied instructions (QuantiTect Whole Transcriptome Kit, QIAGEN, Australia). The cDNA obtained from the amplification process was used as a template in a polymerase chain reaction (PCR) reaction using human beta actin primers (forward: ATC TGG CAC ACC TTC TAC AAT GA; reverse: CGT CAT ACT CCT GCT TGC TGA TCC AC) (Integrated DNA Technologies, NSW, Australia). There was an initial denaturation for 2 minutes at 98°C, followed by 30 cycles of 98°C for 30s (denaturation), 67°C for 30s (annealing), and 72°C for 1 minute (extension). The cDNA and PCR products were run on a 1% agarose gel (Bio-rad Laboratories, Inc., Australia) and visualized with RedSafe (ChemBio, UK). HyperLadder™ 1 kb (HyperLadder I) (Bioline, UK) was used to determine the mass of cDNA and human beta actin amplicons.

### Statistical analysis

Statistical analysis was performed using PRISM 6 for Windows (GraphPad Software, Inc., USA). Channel width, velocity and roughness amplitude data were presented as mean ± SD. Pain score for channel width and velocity were presented as minimum to maximum box-and-whiskers plot. One-way ANOVA combined with a Tukey’s multiple comparison post-test was performed to determine the statistical significance.

## Results

Our microbiopsy device has a chamber volumetric size of 0.003 mm ^3^ that is more than 6000 times smaller than a conventional 3 mm punch biopsy and more than 5 times smaller than an 18 GA syringe needle ( Figure 1a). Figure 1b demonstrates the size differences between a conventional 3 mm punch biopsy (top panels) and our microbiopsy (bottom panels).

### Channel width optimisation

**Figure 2.**
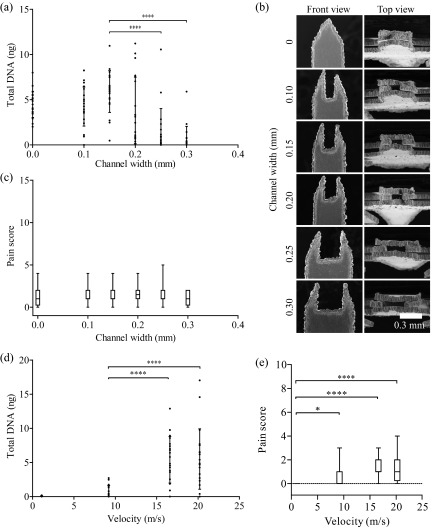
Microbiopsy channel width and velocity optimisation. Channel width and velocity were varied to optimise the microbiopsy device configuration. Total DNA was used as a surrogate for sample size. Panel ( **a**) shows the DNA extracted from device with varying channel width and that the maximum amount of DNA was collected with 0.15 mm channel width. Panel ( **b**) displays high resolution scanning electron microscopic images showing different channel widths of the microbiopsy device. Panel ( **c**) shows the level of pain volunteers reported when different channel width microbiopsies were used. Panel ( **d**) shows the varying velocity applied and that the maximum amount of DNA was collected when the device was applied at 16.6 m/s. Panel ( **e**) shows the level of pain volunteers reported when application velocity was varied.

We conducted a volunteer study in 20 healthy individuals to determine the optimal channel width and application velocity for this device. Extracted DNA mass was chosen to be the primary indicator for sample-to-sample comparisons. Interestingly, we observed tissue collection (4.5 ± 1.5 ng DNA) around the rough edges of a microbiopsy device without a chamber ( Figure 2a–b, channel width of 0 mm). After applying the microbiopsy, the device was opened up and visualized under a dissecting microscope. Successful collection was achieved when tissue was evident within or around the device and unsuccessful if no tissue was present. Tissue was collected from all volunteers (n=20) when a 0.15 mm channel width microbiopsy device was used. Only 13 successful collections were achieved from 20 applications when a 0.20 mm channel width microbiopsy device was used. This indicated that the collection rate decreased from 100% to 65% when channel width was increased by 0.05 mm. The device without a channel (0 mm channel width) captured tissue around the edges of the tapered plates in all replicates (n=20). This experiment showed that a channel width of 0.15 mm obtained the highest average amount of DNA (5.9 ± 3.4 ng), which was significantly higher than 0.25 and 0.30 mm channel widths (p < 0.0001). There was no significant difference in the total DNA extracted between 0–0.20 mm channel widths ( Figure 2a).

### Application velocity optimisation

Microbiopsy application velocity was evaluated by using defined applicator springs to achieve velocities between 0–20.2 m/s ( Figure 2d). Only negligible amounts of DNA were recovered when the device was applied at less than 9.2 m/s. However, there was a 7.5-fold increase (0.8 ± 0.8 to 6.0 ± 3.0 ng) in DNA recovered when the application velocity was increased from 9.2 m/s to 16.6 m/s (p < 0.0001). An additional increase to 20.2 m/s increase in application velocity did not result in significant increases in DNA collection.

### Pain scale assessment

There was no change in the level of pain reported when microbiopsies with varying channel widths were applied ( Figure 2c). The level of pain significantly increased and variation increased when microbiopsies were applied at increasing velocities ( Figure 2e). All of the volunteers had a pain score of 0 at 5 minutes after the final microbiopsy application (data not shown). The volunteers scored pain between 0 to 10 with an average score of 1.5 ± 1.1, when the 0.15 mm channel width microbiopsy was applied at 20.2 m/s.

### Edge roughness optimisation

**Figure 3.**
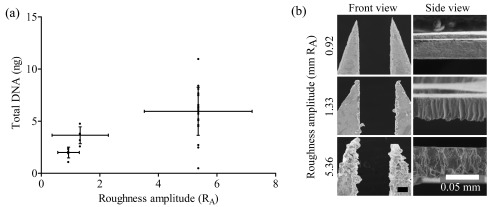
Microbiopsy roughness amplitude optimisation. Panel ( **a**) shows that microbiopsy devices with higher roughness amplitude channels are capable of collecting more DNA. Panel ( **b**) contains high resolution scanning electron microscopic images showing different channel widths and roughness of the microbiopsy device.

We observed DNA collection without a centre chamber ( Figure 2a, 4.5 ± 1.4 ng from channel width 0 mm) and hypothesized that surface roughness could be key to successful sample collection. We compared microbiopsy devices with varying roughness amplitudes (R _A_) and observed an increasing trend in total DNA extracted with increasing R _A_ ( Figure 3a) (n=20 for 5.36 R _A_ and n=5 for 0.92 and 1.32). The SEM images of the inner chamber edge were used to measure the R _A_. These measurements ranged from relatively smooth (R _A_ 0.92) to rough (R _A_ 5.36) ( Figure 3b, side view).

### Microbiopsy site imaging

**Figure 4.**
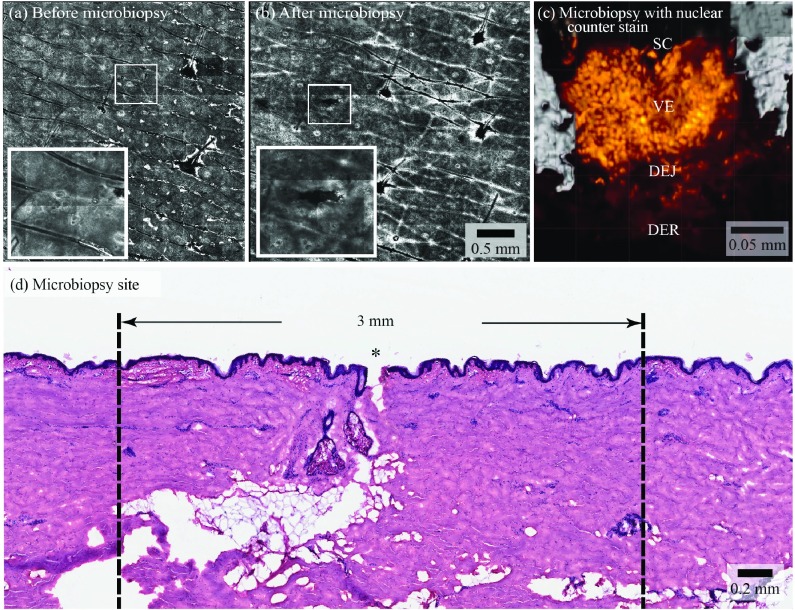
Site of microbiopsy and microbiopsy content. Panels ( **a**) and ( **b**) are reflectance confocal microscopy mosaics of a microbiopsy site, see the hair follicles featured in the centre and on the right hand side of the images for size comparison (bar indicates 0.5 mm in **a** and **b**). Panel ( **c**) shows a 63x magnification, 3D rendering of the microbiopsy tissue with a nuclear counter stain (orange) derived from a confocal microscopy z-stack of the sample within the microbiopsy device. The stratum corneum (SC), viable epidermis (VE), dermal-epidermal junction (DEJ) and superficial dermis (DER) are labeled. This microbiopsy contained an estimated 1634 nuclei. Haematoxylin and eosin stained section of human skin after microbiopsy application shows a 0.10 mm wide and 0.25 mm deep puncture Panel ( **d**). * indicates the site of microbiopsy application.

RCM was used to visualise the microbiopsy application site. RCM images revealed a microbiopsy site similar to the size of a small hair follicle in the dermal papillary post microbiopsy application ( Figure 4a and 4b). The inset shows that microbiopsy application resulted in a puncture site that was approximately 0.10 × 0.50 mm in dimension ( Figure 4b). Microbiopsy samples were stained with DRAQ5 (BioStatus Limited, UK) to highlight the nuclei. Confocal images were used to generate a 3D model of the microbiopsy sample. The usual skin strata were apparent in this model. We estimated that there were 1634 nuclei in Figure 4c. We observed 0.22 ± 0.12 mm wide and 0.26 ± 0.09 mm deep puncture sites in excised abdominal skin from 10 histological sections ( Figure 4d).

### Microbiopsy sample molecular characterisation

**Figure 5.**
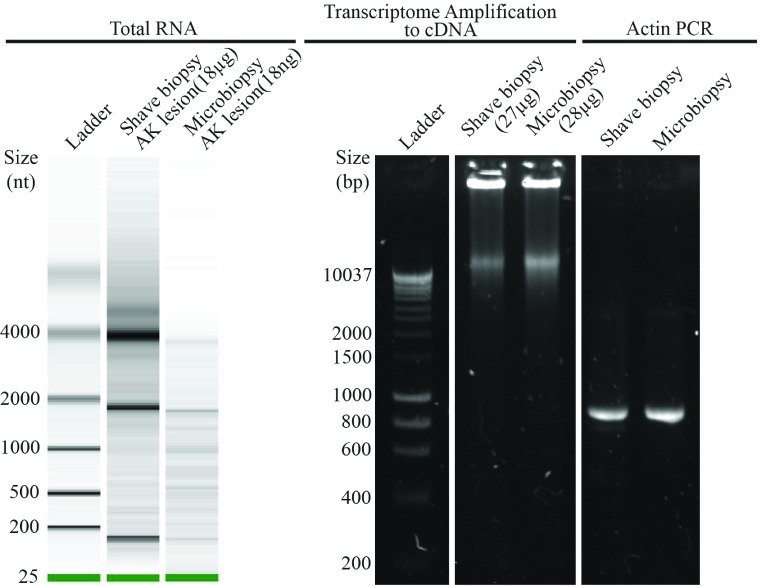
Molecular characterization of conventional shave biopsies and microbiopsies. The left panel shows the Bioanalyzer readout from amplified DNA from a conventional actinic keratosis (AK) shave biopsy next to the microbiopsy DNA sample obtained from the same AK lesion. The middle panel shows the Bioanalyzer readout from RNA isolated from a conventional AK shave biopsy and a microbiopsy that was obtained from the same AK lesion. The right panel compares the RNA quality of a shave biopsy and normal skin microbiopsy that were subjected to total transcriptome amplification to generate cDNA. The cDNA was then used as a template for a PCR reaction containing actin specific primers with an expected product at 800 bp.

One of our goals was to compare AK lesions to microbiopsy samples since many of these lesions can be present in patients but only a few progress ^12^ to squamous cell carcinoma and the molecular mechanism behind this transition is a focus of intense research. Microbiopsy samples were taken from freshly excised AKs. The RNA from these matched samples had comparable quality with an average RIN difference of -0.85 ± 0.85 for n=4. Representative Bioanalyzer (Agilent Technologies, USA) results for the conventional biopsy (RIN 6.50) and microbiopsy (RIN 5.10) are shown in Figure 5, left panel. The results showed that there are similarities and differences in the RNA bands, which may be due to the large amount of tissue sampled with conventional biopsy and the relatively small number of cells sampled with the microbiopsy.

The small sample size is an inherent limitation of the microbiopsy device. To mitigate this, a pilot experiment was conducted where the microbiopsy sample was subjected to whole transcriptome amplification for RNA analysis. We included a skin sample with the same amount of starting material (14 ng) as an amplification control in the experiment. We observed a 2000-fold increase in cDNA from both samples based on total RNA and cDNA measurements. The cDNAs were of comparable quality and quantity ( Figure 5, Transcriptome Amplification to cDNA). Subsequently, we used PCR to amplify human beta-actin mRNA. We observed 2 identical bands at 800 bp with comparable PCR product quality both lanes ( Figure 5, Actin PCR).

### Wound healing kinetics

**Figure 6.**
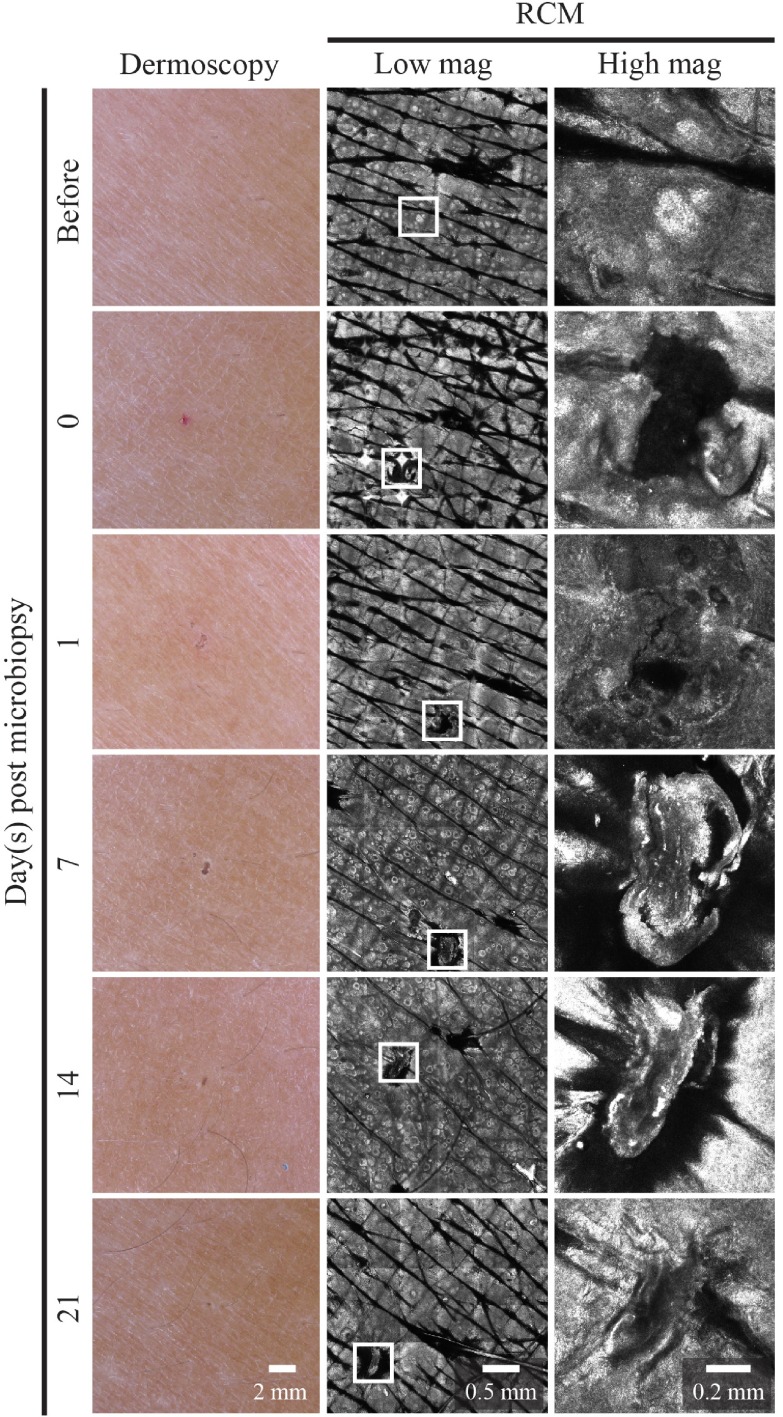
Wound healing kinetics of microbiopsy site. The left column shows dermoscopic images of the microbiopsy site over time. The middle and right column are mosaics and at 30x magnification reflectance confocal microscopy (RCM) images, respectively, of the microbiopsy site.

Immediately after microbiopsy application we observed local erythema that resolved within 24 hours. The tiny excision site from the microbiopsy healed quickly and was invisible to the naked eye after 24 hours ( Figure 6). We used dermoscopy and RCM to monitor the wound healing process at regular intervals ( Figure 6, center and right columns).

Influence of channel width and velocity of microbiopsy on DNA, extraction, RNA extraction and pain scores in volunteers Channel width DNA extracted: total DNA extracted (ng) from different channel widths (mm) in 20 volunteers (v1-v20).
Channel width pain scores: the level of pain scored (10 point Likert scale) by 20 volunteers (v1-v20) when applied with different channel widths (mm).
Velocity DNA extracted: total DNA (ng) extracted using microbiopsy at different velocities (m/s) in 20 volunteers (v1-v20).
Velocity pain scores: the level of pain (10 point Likert scale) scored by 20 volunteers (v1-v20) in response to different velocities (m/s).
Roughness amp. DNA extracted: the total DNA (ng) extracted using microbiopsy with different roughness amplitude in 20 volunteers (v1-v20).
RNA extracted: the total RNA extracted (ng) from excised AK lesions using 0.15 mm channel width microbiopsies (n=5).

## Discussion and conclusion

Whereas conventional biopsies allow accurate diagnosis of tissue sampled through histopathological assessment, there is a need for an alternative technology, such as the microbiopsy, to screen multiple lesions through molecular diagnosis. In this proof of concept study, we have shown that an arrangement of stacked plates to form a 3D microbiopsy device is capable of providing molecular samples from normal and diseased skin. DNA extraction from human skin is critical for stratifying lesions in terms of mutational status. This type of characterization is becoming more and more relevant as targeted signalling inhibitors are being added to the therapeutic arsenal. Recent developments in molecular inhibitors for skin cancer, e.g. Vemurafenib ^13^ and Trametinib ^14, 15^, are driving a new need for molecular diagnostic information that cannot be gathered through morphologic analysis. Molecular biomarkers have been used to detect cancer, but are now being developed to determine which molecular therapeutic will be the most effective. Melanoma clinical biology research has resulted in genomic data identifying oncogenes that can be targeted by protein kinase inhibitors e.g. *BRAF* and *MEK* ^16^. Diagnostic assays to detect *BRAF* and *NRAS* mutations are now being used to stratify skin cancer lesions ^17^. The microbiopsy device has the potential to help screen multiple lesions for specific mutations, and to be important in discovery based research.

Yancovitz *et al.* describe intra- and inter-tumour heterogeneity of melanoma in the context of *BRAF ^V600E^* mutations ^18^. Hematoxylin and eosin stained sections of primary and metastatic lesions were subjected to laser capture microdissection to isolate the lesions of interest. They captured 30–300 cells for each lesion of interest. This is less than 5 times that captured with the microbiopsy device. The authors used the same DNA extraction kit as we did to isolate lesional DNA for analysis. These samples were then used to detect *BRAF ^V600E^* tumour heterogeneity. Based on our analysis, we estimate that Yancovitz *et al.* isolated <1 ng of DNA in this study and were able to amplify *BRAF* exon 15 using conventional PCR followed by mutation analysis. We found that we can reproducibly isolate 5 ng of DNA from the superficial skin and in another publication show the use of the microbiopsy device in dysplastic nevi ^11^. This work sets the stage for future microbiopsy studies focused on *BRAF* mutational analysis in nevi *in situ*. The addition of RNA analysis to mutational studies would give additional insights into gene expression profiles of these lesions.

Berglund *et al.* (2007) reported that their optimised methodology yielded an average 1.4 ± 0.4 µg of consistently high quality RNA (8.4–8.9 RIN) from 3 mm skin punch biopsies ^19^. Another group reported that they had isolated an average of 1.5 µg of RNA with an average RIN value of 8.1 from half of a 4 mm punch biopsy skin sample (n=97) ^20^. The average total RNA yielded from the microbiopsy device is 9.0 ± 10.1 ng (n=5). Even though we isolated far less RNA with the microbiopsy device, our proof of concept study supports the hypothesis that this limitation can be addressed using commercial amplification kits and PCR ( Figure 5) for investigating the molecular basis of skin disease. Through the course of these experiments we observed RNA integrity values that ranged between 1.2 and 7.6 with the microbiopsy. We also observed that these values correlated with matched conventional shave biopsies. In some cases the values were quite low (e.g. RIN 5.1 in Figure 5) and would not be considered for whole transcriptome approaches. Sampling for RNA analysis can be difficult and optimizing this application for the microbiopsy is one of our priorities as we move forward with this technology.

Discovering non-melanoma skin cancer biomarkers and potential therapeutic targets is an emerging area of research. For example, Dang *et al.* (2006) focused on the genetic changes that occur in AK to squamous cell carcinoma (SCC) for prospective development of new diagnostic tools and therapeutic approaches. They reported that 7 out of 14 genes in 10 AK, 10 SCC and 20 normal skin samples related to cell adhesion, communication, metabolism and respiration were significantly dysregulated in AK and SCC (p < 0.05) ^21^. The microbiopsy device has the potential to help facilitate longitudinal studies within a single lesion.

Application of the relatively small microbiopsy device does not require local anaesthesia or sutures and therefore no set up for a minor clinical procedure is necessary. Observations from our volunteer study suggest that collecting multiple microbiopsies within in a short period is feasible. We envision that microbiopsy devices will become a routine clinical and research device in dermatology allowing the dermatologist to obtain targeted lesion data for molecular stratification.

## Consent

Written informed consent for publication of their clinical images was obtained from the patients (HREC/11/QPAH/477 and/or HREC/12/QPAH/082, Metro South Human Research Ethics Committee, Centres for Health Research, Princess Alexandra Hospital).
