# Supplementary material for: Microbiopsy engineered for minimally invasive and suture-free sub-millimetre skin sampling
Source: F1000Res. 2013 Jul 31;2:120. Originally published 2013 May 2. [Version 2] doi: 10.12688/f1000research.2-120.v2 (PMC3907159; doi:10.12688/f1000research.2-120.v2)
Supplement: Peer review history

# Peer review: Referee response for version 2

**Published:** 2013-08-07

**Authors:** Jack Arbiser

**Affiliations:** 1 Winship Cancer Insitute, Emory University, Atlanta, GA, USA

I am satisfied with the revisions made by the authors and these changes have improved the article.

I have read this submission. I believe that I have an appropriate level of expertise to confirm that it is of an acceptable scientific standard.

# Peer review: Referee response for version 2

**Published:** 2013-07-31

**Authors:** Marco Ardigò

**Affiliations:** 1 Dermatology, San Gallicano Dermatological Institute, Rome, Italy

I have read this submission. I believe that I have an appropriate level of expertise to confirm that it is of an acceptable scientific standard.

# Peer review: Referee response for version 1

**Published:** 2013-06-21

**Authors:** Neil Rajan

**Affiliations:** 1 Newcastle University, Newcastle, UK

This is an interesting study, describing the development and characterisation of a device to take small biopsies from skin lesions. Attractively, it appears to allow for sampling of tissue with minimal discomfort. The extracted sample can be processed through DNA and RNA extraction protocols. The authors describe how this device may influence future changes in molecular diagnostics in skin tumours. 

The paper is well written. There are a few minor revisions that should be made for it to be acceptable for indexing. 

• **Title and Abstract:**

The word painless in the second line should be removed as this is not substantiated by the data provided. The volunteers expressed that pain was experienced at the time of the biopsy, indicated in the score in Table 1. 

• **Article content:**

*Improved wound healing at high risk sites*

Page 3 of 11 –  “Certain areas such as the lower leg may have problems with wound healing and so biopsies are avoided. The microbiopsy device fills a void in dermatology where conventional biopsies are not feasible or could do more harm than good.” – This needs to be substantiated by data showing that the device resulted in fewer lower leg complications for this statement to stand. Otherwise it should be removed.

*Comparison to 4mm punch biopsies*

The disadvantages of the punch biopsy approach (anaethesia, suturing) are highlighted. Whilst the thrust of the article is the extraction of tissue for molecular analysis at a nucleic acid level, for a balanced view, it should be pointed out that punch biopsies allow for histological assessment of the tissue sampled, whilst the microbiopsy device does not. 

It may also be worth considering future studies where pain scores are assessed in patients who volunteer to have both a punch biopsy and a microbiopsy undertaken, to compare the difference between the 2 approaches.

*RNA quality*

It has to be acknowledged that whilst the RNA extracted was amplified to cDNA, the starting RNA integrity number of the sample published (5.5) would not be considered suitable for whole transcriptome approaches, such as RNA –seq and microarray profiling, where ideally a RIN of >8 is desirable. 

*DNA quality*

It would be good to see an indication of the DNA quality and integrity from tissue extracted with this device, using either a bioanalyzer approach or an agarose gel.

**•    Conclusions:**

The conclusions are sensible, balanced and justified on the basis of the results of the study.

I have read this submission. I believe that I have an appropriate level of expertise to confirm that it is of an acceptable scientific standard.

# Peer review: Referee response for version 1

**Published:** 2013-06-19

**Authors:** Marco Ardigò

**Affiliations:** 1 Dermatology, San Gallicano Dermatological Institute, Rome, Italy

This is an interesting, innovative and well conducted study about the use of microinvasive (“quasi” on invasive) tissue sampling for confocal-molecular correlation performed with the new method of the sub-millimeter punch. 

Title and abstract are appropriate.

Conclusions are justified on the basis of results of the study.

In conclusion, the study really increases the chances of routine applications of micro-punches for tissue sampling for targeting molecular analysis in skin diseases.

I have read this submission. I believe that I have an appropriate level of expertise to confirm that it is of an acceptable scientific standard.

# Peer review: Referee response for version 1

**Published:** 2013-06-19

**Authors:** Jack Arbiser

**Affiliations:** 1 Winship Cancer Insitute, Emory University, Atlanta, GA, USA

The title and abstract, article content, conclusions and data are all highly appropriate. I am particularly impressed with the ability to obtain intact DNA and RNA from this technique. Given the almost total lack of scarring, I predict that this method will be highly used and this paper will be highly cited in the field. I look forward to utilizing it in my own practice. The ease of wound healing makes it particularly attractive

I have read this submission. I believe that I have an appropriate level of expertise to confirm that it is of an acceptable scientific standard.
